# Glycosaminoglycan Neutralization in Coagulation Control

**DOI:** 10.1161/ATVBAHA.118.311102

**Published:** 2018-05-29

**Authors:** Amélie I.S. Sobczak, Samantha J. Pitt, Alan J. Stewart

**Affiliations:** From the School of Medicine, University of St Andrews, Fife, United Kingdom.

**Keywords:** dermatan sulfate, glycosaminoglycan, heparan sulfate, heparin, thrombosis

## Abstract

The glycosaminoglycans (GAGs) heparan sulfate, dermatan sulfate, and heparin are important anticoagulants that inhibit clot formation through interactions with antithrombin and heparin cofactor II. Unfractionated heparin, low-molecular-weight heparin, and heparin-derived drugs are often the main treatments used clinically to handle coagulatory disorders. A wide range of proteins have been reported to bind and neutralize these GAGs to promote clot formation. Such neutralizing proteins are involved in a variety of other physiological processes, including inflammation, transport, and signaling. It is clear that these interactions are important for the control of normal coagulation and influence the efficacy of heparin and heparin-based therapeutics. In addition to neutralization, the anticoagulant activities of GAGs may also be regulated through reduced synthesis or by degradation. In this review, we describe GAG neutralization, the proteins involved, and the molecular processes that contribute to the regulation of anticoagulant GAG activity.

Heparan sulfate (HS), dermatan sulfate (DS), and heparin are natural glycosaminoglycans (GAG), which are linear polysaccharides, heterogeneous in both sequence and length.^1^ GAGs carry out many functions in the body and can influence numerous physiological processes. The most notable is control of coagulation, but GAGs also affect lipid metabolism, inflammation, cell attachment, migration, invasion, and differentiation.^1^ GAGs play a key role as anticoagulants, preventing coagulation from occurring when it is not required. GAGs are responsible for interacting with and enhancing the actions of several serpins; HS and heparin primarily bind to antithrombin and DS to heparin cofactor II (HCII).^2^ However, HS and heparin can also interact with HCII.^3^ The principal activities of antithrombin are to inhibit both thrombin and activated factor X, 2 important proteins of the coagulation cascade.^2^ HCII inhibits thrombin but not activated factor X.^3^

When coagulation is necessary, for example, after tissue injury, GAGs need to be neutralized to enable clot formation.^4^ This includes endogenous GAGs during normal clotting and heparin-based drugs, which are used clinically to treat a range of thrombotic disorders, including venous thromboembolism and acute coronary syndrome.^5^ This review will describe current knowledge concerning the principal properties of key proteins involved in GAG neutralization, the mechanisms by which they interact with GAGs, and how this affects the coagulation process.

## Synthesis of GAGs

**Figure 1.**
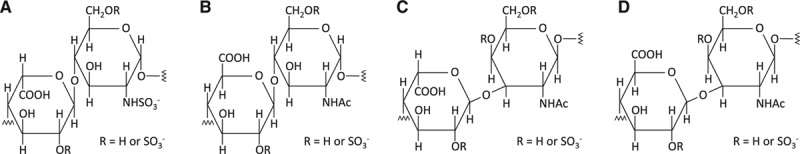
The principal disaccharide motifs that constitute heparin, heparan sulfate, and dermatan sulfate. **A**, Heparin and heparan sulfate motif 1, iduronic acid-*N*-sulfated glucosamine. **B**, Heparin and heparan sulfate motif 2, glucuronic acid-*N*-acetylated glucosamine. **C**, Dermatan sulfate motif 1, iduronic acid-*N*-acetylated galactosamine. **D**, Dermatan sulfate motif 2, glucuronic acid-*N*-acetylated galactosamine.

The saccharide sequences of HS and heparin consist predominantly of 2 trisulfated disaccharide motifs. The first motif represents *N*-sulfated glucosamine linked to iduronic acid (IdoA) and the other *N*-acetylated glucosamine (GlcNAc) linked to glucuronic acid (GlcA).^1^ The sequence in which these motifs occur results from enzyme-catalyzed modifications.^6–8^ Heparin and HS differ in the ratio by which these 2 motifs are present within the GAG; heparin is defined as containing at least 70% of the first motif.^8^ The sequences of DS consist of 2 different motifs. These are *N*-acetylated galactosamine (GalNAc) linked to IdoA and GalNAc linked to GlcA.^1^ The sequences of the saccharide groups that form HS, heparin, and DS are shown in Figure 1.^1^ Some GAGs carry specific binding sequences for antithrombin and HCII, and those can greatly enhance the efficiency of the binding to those serpins.^3,9,10^

The length of the GAGs, sulfation percentage, and saccharide sequence vary depending on the tissue in which they are generated because of cell type–specific expression of GAG-synthesizing enzymes (as summarized in Refs. Carlsson and Kjellen^9^ and Silbert and Sugumaran^11^). Synthesis begins by formation of a tetrasaccharide, GlcA–galactose–galactose–xylose, which forms the linkage region. For HS and heparin, the next saccharide to attach is GlcNAc, which is performed by a unique GlcNAc transferase-I enzyme that plays no further part in the synthesis. For DS, it is a GalNAc transferred by a GalNAc transferase enzyme. The chain is then further elongated by transferase enzymes, which add alternating GlcA and either GlcNAc or GalNAc. As the GAG chain grows, it is modified by the action of various enzymes, which include (1) the *N*-sulfation of GlcNAc to *N*-sulfated glucosamines by *N*-deacetylase/*N*-sulfotransferase; (2) the epimerization of D-GlcA saccharides, adjacent to *N*-sulfated glucosamines or GalNAc 4-S, to L-IdoA by C5-epimerases; (3) the 6-*O*-sulfation of GlcNAc and GalNAc by 6-*O*-sulfotransferases; (4) the 4-*O*-sulfation of GalNAc by 4-*O*-sulfotransferases; and (5) the 2-*O*-sulfation of IdoA (and in to a lesser extent GlcA) by 2-*O*-sulfotransferases.

**Figure 2.**
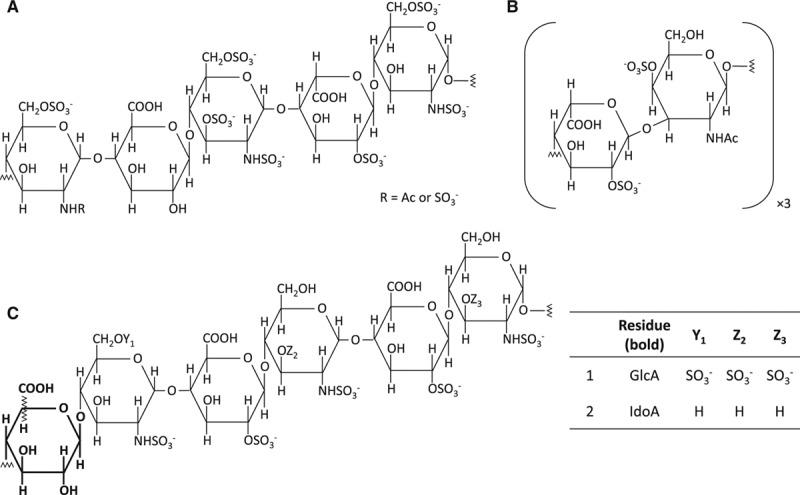
Anticoagulant glycosaminoglycans-binding sequence for antithrombin and heparin cofactor II. **A**, The main heparin and heparan sulfate sequence for binding to antithrombin. **B**, The main dermatan sulfate sequence for binding to heparin cofactor II. **C**, Two heparin and heparan sulfate sequences for binding to heparin cofactor II. The sequences were predicted in silico, and their ability to neutralize those two glycosaminoglycans was confirmed in vitro.^10^ GlcA indicates glucuronic acid; and IdoA, iduronic acid.

In addition, a less frequent but important modification is the sulfation in the C3 position of GlcNAc by 3-*O*-sulfotransferase, which forms part of the antithrombin-binding site.^9^ This antithrombin-binding site on HS and heparin is a pentasaccharide sequence. On DS, the HCII-binding sequence is an hexasaccharide sequence consisting of a repeat of IdoA(2-OSO_3_^−^)-GalNAc(4-OSO_3_^−^).^3^ The natural binding sequences for HCII on HS and heparin have not yet been confirmed, but 2 possible HS hexasaccharide structures have been predicted based on an in silico study, and enhancement of HCII inhibition of thrombin has been confirmed after synthesis of the hexasaccharides.^10^ The serpin-binding sites on HS, DS, and heparin are shown in Figure 2.

## Distribution of HS, DS, and Heparin and Their Anticoagulant Actions

HS and DS are synthesized by many cell types and tissues, whereas heparin is only synthesized in mast cells.^12^ HS is mainly localized at the surface of cells and the endothelium,^1^ while DS is present in the extracellular matrix of several types of tissue including skin, bone, cartilage, and the vasculature.^1^ The surface of the endothelium is made of a layer of glycoproteins and proteoglycans called the endothelial surface layer (ESL), which is made of a core protein bound to one or several GAG chains. The ESL varies in thickness depending on its location: ranging from around 0.5 to 3 μm in small arteries ≤4.5 μmol/L in carotid arteries.^13^ ESL thickness is also influenced by oxidative stress and atherosclerosis.^13^ The composition of the ESL is dynamic; ESL proteins undergo a high rate of turnover, and the specific GAGs that are present (and their sulfation pattern) also change over time. Turnover and GAG-binding specificity depends on the activation of the endothelial cells by local chemokine stimuli.^13^ A summary of proteoglycans present in the ESL and their GAG-binding properties are provided in Table 1.

**Table 1.**
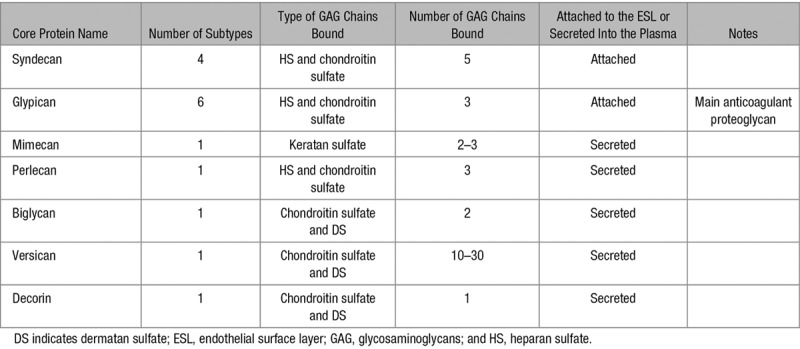
Proteoglycans Present in the ESL and Their GAG-Binding Properties^13^

**Figure 3.**
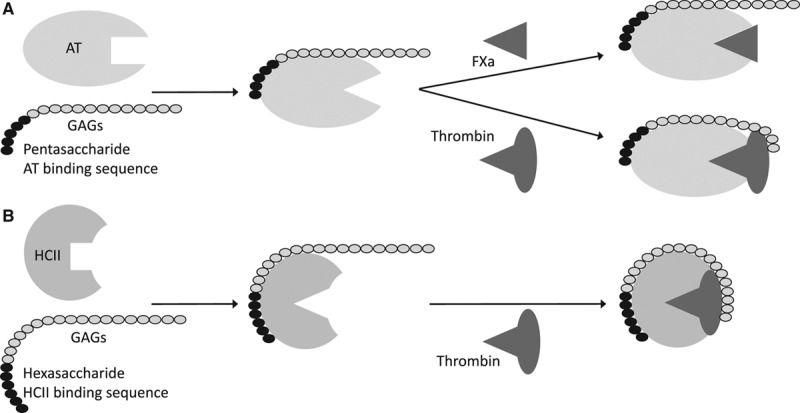
Interactions of glycosaminoglycans (GAGs) with (**A**) antithrombin (AT), thrombin, and activated coagulation factor X (FXa) and (**B**) heparin cofactor II (HCII) and thrombin. The GAGs bind AT and HCII via specific binding sequences, a pentasaccharide or a hexasaccharide sequence, respectively. This binding induces a conformational change in the serpins, which makes the reactive center loop more accessible for their substrates. A simultaneous binding to AT and thrombin requires a chain length of minimum 18 saccharides while binding to HCII, and thrombin requires 24 saccharides.

An important aspect of the ESL is its anticoagulant properties. Indeed, ESL GAGs bind several anticoagulant proteins, antithrombin, HCII but also thrombomodulin and tissue factor pathway inhibitor. The endothelial anticoagulant HS, for example, is saturated with antithrombin (the *K*_d_ for this interaction is 15 nmol/L, while antithrombin plasma concentration is 3.5 μmol/L).^14^ GAG binding to serpins induces a change in the conformation of the reactive center loop of the serpin, thus enhancing the inhibitory activity of the protein.^2^ Longer chain GAGs are specifically required to enhance the binding of antithrombin to thrombin but not to activated factor X.^2^ This is also the case for HCII and thrombin, although some thrombin inhibition still occurs in the presence of shorter chain GAGs (Figure 3).^3^

The main GAG in the vasculature is HS, which represents 50% to 90% of the total GAG content.^13^ Next is chondroitin sulfate, of which DS is a subtype.^13^ Although all membrane-associated HS can bind antithrombin, only a small fraction of these molecules (0.5%–10%) possess the antithrombin-binding sequence necessary to bind with high specificity under physiological conditions.^14,15^ Approximately 95% of anticoagulant HS is present in the subendothelial matrix and is only in contact with blood when injury occurs.^14,15^ In the case of heparin, ≈30% of molecules possess the antithrombin-binding sequence.^16^ The exact proportion of HS and heparin that can bind HCII is unknown, but a much greater quantity of the GAG is required to overcome HCII-mediated inhibition than with antithrombin.^3^ Vascular DS is located in the deeper layer of the vessel walls and in the subendothelium and is only able to interact with blood proteins during injury.^3^ Roughly 5% of DS disaccharides are the IdoA(2-OSO_3_^−^)-GalNAc(4-OSO_3_^−^) disaccharide unit, of which 3 consecutive repeats are required to form the HCII high-affinity-binding sequence. Chains containing only part of the total sequence still bind HCII but with a lower affinity.^3^

The importance of heparin release from mast cells at sites of injury is controversial. Some studies have been unable to detect heparin in plasma,^17,18^ while Engelberg and Dudley^19^ (1961) reported that each liter of plasma contains 1.0 to 2.4 mg of heparin (*ca.* 66–160 nmol/L). To ascertain the importance of endogenous anticoagulant GAG in vivo, 2 knockout mice models have been studied. Hemostasis in mice lacking 3-*O*-sulfotransferase-1 (the enzyme involved antithrombin-binding site formation) was not greatly affected by removal of the gene encoding this enzyme, where a strong procoagulant challenge failed to reveal a latent procoagulant state.^14^ This suggests that either the anticoagulant activity of HS is not essential for normal homeostasis in vivo or that there is redundancy between sulfotransferase-1 and one of its isotypes (eg, 3-*O*-sulfotransferase-5). It is also possible that antithrombin expression is increased in these animals to compensate for the loss of the enzyme.^14,20^ In addition, HS lacking the canonical antithrombin-binding sequence can still bind antithrombin but exhibits reduced affinity compared with HS with the binding sequence present. This HS has antithrombin activity but not anti–activated factor X activity.^14^ Mice engineered to lack the *N*-deacetylase/*N*-sulfotransferase 2 enzyme (encoded by the *NDST2* gene), which is involved in heparin, but not HS, synthesis have also been studied.^21,22^ No coagulatory defects were reported in these animals. However, these studies did not specifically examine clot parameters.^18,21,22^

## Use of Heparin-Based Drugs

Clinically, heparins are the main anticoagulants administered for several conditions, including venous thromboembolism, acute coronary syndrome, cardiopulmonary bypass, and hemodialysis.^7^ Naturally occurring heparins vary in size from 3 to 30 kDa, with an average of 15 kDa and when used therapeutically are termed unfractionated heparins (UFH). UFH can be fully neutralized by protamine sulfate when there is a risk of bleeding.^23^ Low-molecular-weight heparins (LMWH) are artificially derived from UFH by depolymerization or fractionation,^6^ and their use has now replaced that of UFH for many clinical applications. LMWHs are associated with fewer side effects (notably reduced prevalence/severity of osteoporosis and heparin-induced thrombocytopenia) and have a more predictable dose–response profile because they associate less with plasma proteins.^5^ They also have a longer half-life and thus require less frequent administration. It should be noted, however, that LMWHs are more expensive, can only be partially neutralized by protamine sulfate, and cannot be cleared from patients having renal insufficiency (and in such cases they will accumulate over time).^4,7^ A summary of US- and EU-approved heparin-based drugs are provided in Table 2. These also include the synthetic analogue of heparin, Fondaparinux—which is based on the antithrombin-binding sequence. This drug is thought to exhibit fewer side effects than LMWH drugs because of having a higher specificity for antithrombin. Importantly, Fondaparinux cannot be neutralized (although injections of recombinant factor VII may be effective to stop bleeding) and can only be cleared by the renal system. As with LMWH, it is therefore contraindicated in patients having renal disease.^5^ Another drug, danaparoid is a mixture of HS, DS, and chondroitin sulfate and is used in some countries for the treatment of heparin-induced thrombocytopenia.^24^

**Table 2.**
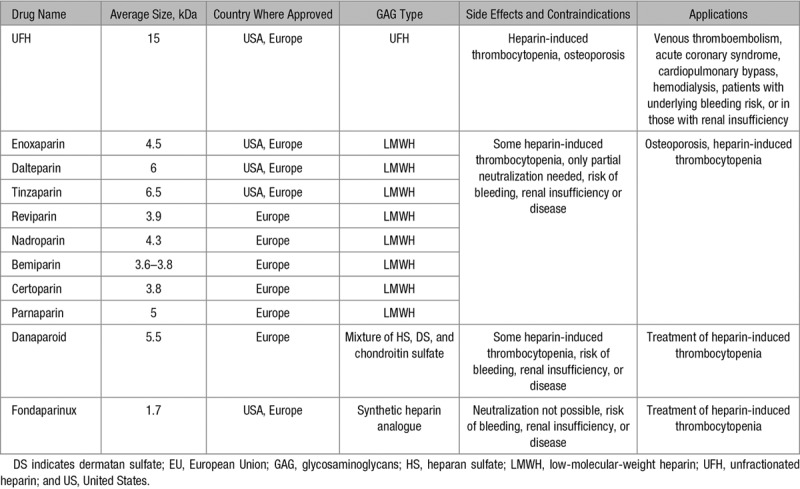
US- and EU-Approved Heparin-Based Drugs, Their Major Side Effects, Contraindications, and Main Applications^5–7,24^

**Figure 4.**
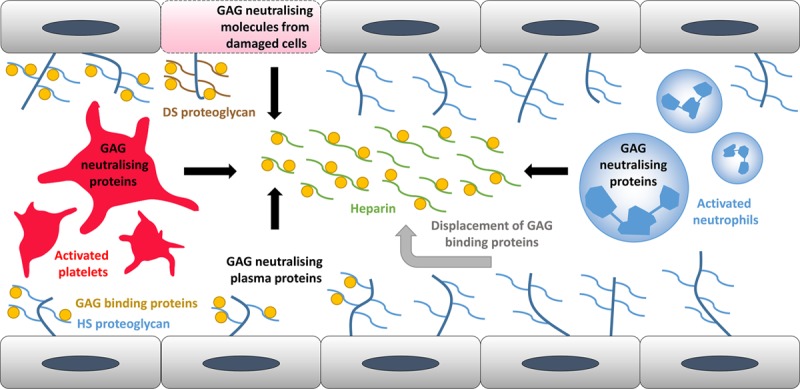
Representation of the key locations where circulatory glycosaminoglycans (GAG)-neutralizing proteins/molecules are derived. Numerous proteins normally bind to endothelial GAGs. They are displaced by injection of heparin. Several key neutralizing molecules are also found in the plasma. Activated platelets and neutrophils can also release such proteins through exocytosis of granular vesicles. Damaged cells expressing dermatan sulfate–containing proteoglycans also release GAG-neutralizing molecules.

The binding of endogenous GAGs to proteins is influenced by various factors, including the degree of sulfation, which facilitates electrostatic interactions between the GAG and the respective protein. Heparin is generally more sulfated than HS, and DS less so.^25^ A possible clinical consequence of such interactions is that administration of heparin-based drugs can potentially displace many of the proteins bound to GAGs in the ESL. This may result in an increase in the availability of GAGs for interaction with clot-regulating proteins (see Figure 4).^26^ Such a mechanism would alter the half-life and kinetic properties of these agents. The associated difficulties in predicting such events could result in excessive bleeding in patients.

Heparin-based drugs are generally best for acute management and prophylaxis of deep vein thrombosis and pulmonary embolism and for prophylaxis during and after orthopedic and general surgeries.^5,27,28^ Current NICE guidelines from the United Kingdom suggest that, when prophylaxis is required, offering patients either Fondaparinux or LMWH (no distinction is made between the different LMWHs) is usually best practice. However, UFH, despite its associated side effects, can be advised when rapid intervention is needed, in individuals with an increased risk of bleeding or those who are having renal failure.^28^ Whether LMWH or UFH are administered for venous thromboembolism prophylaxis is highly variable, and patients have not always received the recommended treatment. The 2007 study—IMPROVE (the International Medical Prevention Registry on Venous Thromboembolism), which examined hospitalized patients at risk of venous thromboembolism across 12 countries—found that out of all patients who should have received prophylaxis (in accordance to the guideline recommendations at the time from the American College of Chest Physicians), 14% of patients in the United States received LMWH and 21% UFH. This preference is because of UFH being a lower cost drug. Across the other participating countries, where downstream costs were more likely to be considered, 40% of patients on average received LMWH and 9% UFH.^29^ From this, it is clear that practices for administration of prophylaxis are suboptimal, and stricter evidence-based guidelines in hospitals urgently need to be implemented.

## GAG-Neutralizing Proteins Released During Injury and Coagulation

The ESL is a heterogeneous surface that can bind proteins and other molecules and is essential for the function of the endothelium.^13^ GAGs associated with the ESL are involved in numerous physiological processes: coagulation, lipid metabolism, inflammation, cell attachment, migration, invasion, and differentiation.^1^ Receptors, enzymes, and their respective ligands/substrates can bind to vascular GAGs, causing a localized rise in their concentration to impact on signaling or enzymatic modification. Fibroblast growth factors (FGF) notably need to bind to endothelial HS for functioning as this helps mediate FGF oligomerization, binding of FGFs to their cognate receptors, and transport of FGF between cells and can act as an FGF reservoir.^30^ Proteins involved in regulating a variety of physiological processes have the ability to neutralize the anticoagulant activity of certain GAGs. A range of these proteins and their specific properties are listed in Table 3. Neutralization can be accomplished via different mechanisms, and so particular neutralizing proteins may affect only certain GAG–serpin combinations (as described in Table 4). Some of these neutralizing proteins are present at high levels in blood plasma, but other common sources include activated platelets, activated neutrophils, and damaged cells (Figure 4). As important binding partners of GAGs, some growth factors have GAG-neutralizing properties. This is the case for FGF7, heparin affin regulatory peptide and, to a lesser extent, FGF1.^31,32^ FGF7 and heparin affin regulatory peptide are also upregulated during injury, thus giving GAGs a dual role in coagulation mediation and wound healing.^32,33^

**Table 3.**
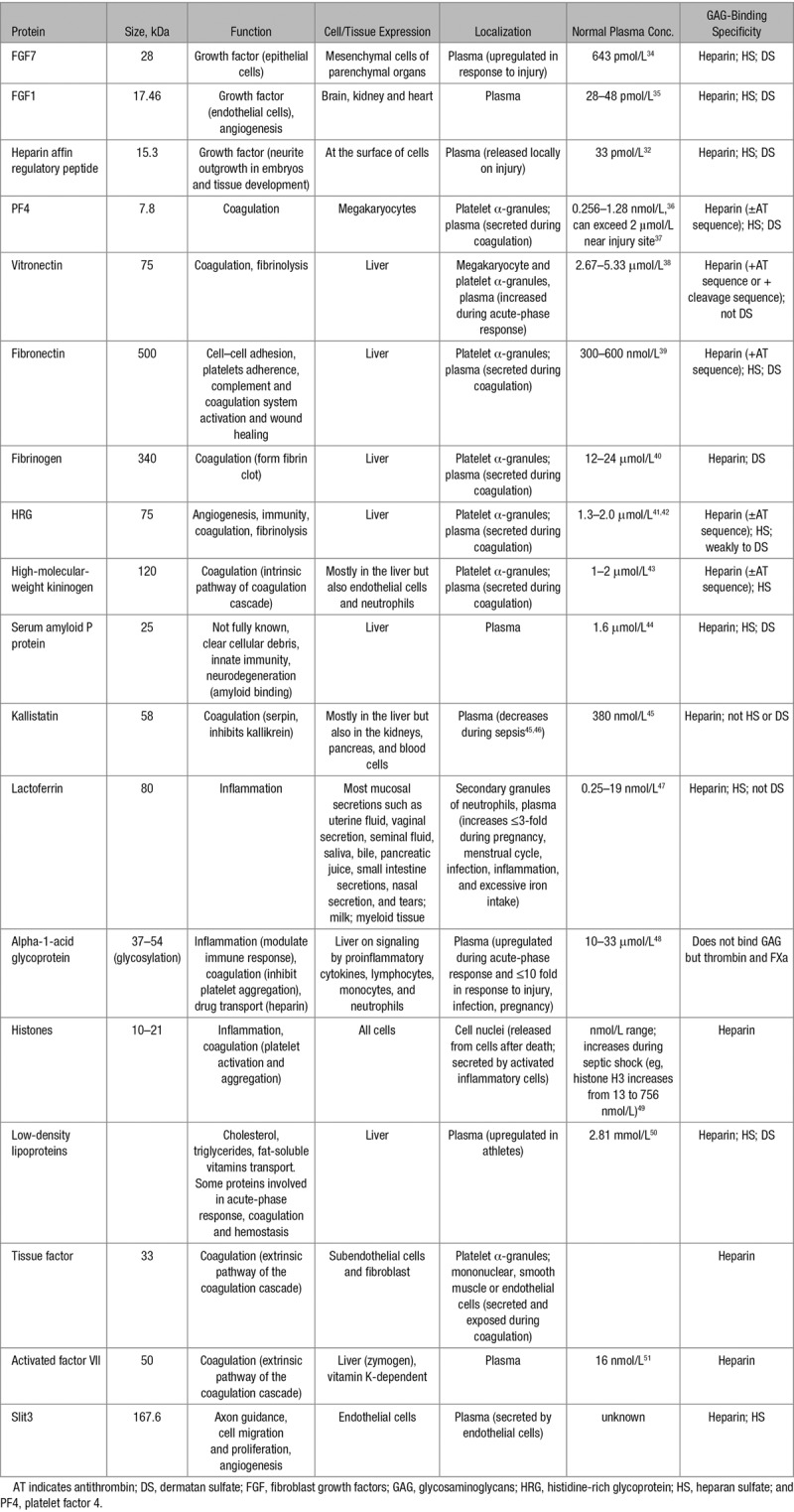
General Properties of Important GAG-Binding Proteins Including Their Functions, Where They Are Synthesized and Stored, When They Are Released, Their Plasma Concentration, and Specific Information on the Abilities of These Proteins to Bind Particular GAGs

**Table 4.**
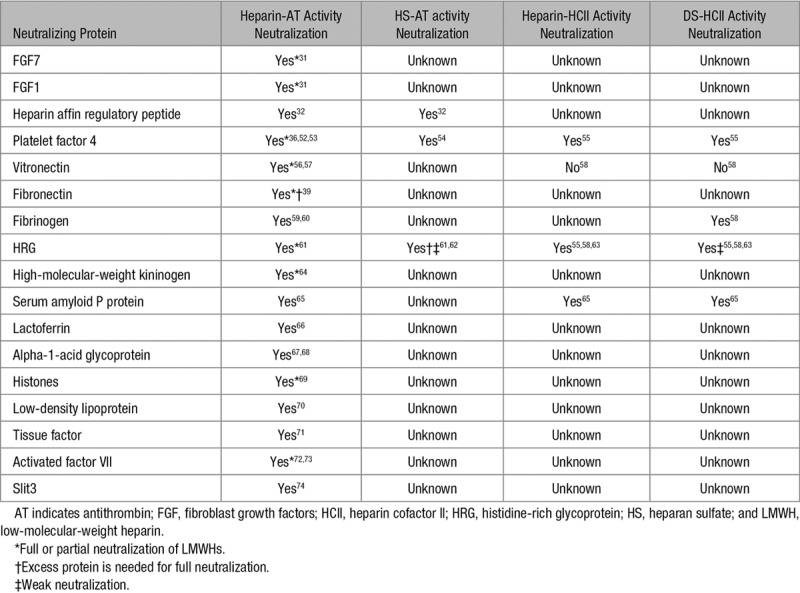
Combinations of GAGs and Serpins Neutralized by Specific Anticoagulant GAG-Neutralizing Proteins

Similar to FGF, chemokine and cytokine activity is directly linked to their ability to bind endothelial GAGs which can direct and enhance their actions. For example, GAGs can modulate the inflammatory response by binding cytokines and so preventing them from binding to cell surface receptors. Cleavage of those GAGs during inflammation releases cytokines which in turn increases endothelial cells activation.^13^ Such chemokines also reside in the α-granules of platelets and are released when these cells are activated. Their GAG-binding ability has been linked to a dual role of GAG in wound healing. Among those is platelet factor 4 (PF4), an important GAG-neutralizing protein. It has been proposed that, when released, PF4 neutralizes the negative charge of GAGs at the surface of endothelial cells. This allows platelets (which possess a net negative charge at their surface) to associate with the endothelium to enhance thrombus formation.^52^ In addition, PF4 binds to nucleic acid (another type of polyanion) exposed by damaged cells. Heparin-induced thrombocytopenia, one of the significant secondary effects caused by heparin administration, is induced by antibodies reacting to the presence of PF4–nucleic acid complexes.^75^

Another family of GAG-neutralizing proteins are proteins involved in coagulation that are also secreted by activated platelets at site of injury. Among those are vitronectin, fibronectin, and fibrinogen. Vitronectin is an abundant plasma protein, and it is released from platelets during the acute-phase response.^38^ It is involved in the regulation of the coagulation, complement, and fibrinolytic systems, as well as that of cell differentiation, proliferation, and morphogenesis.^76^ Vitronectin is also an anticoagulant GAG neutralizer.^77^ The main consequence of GAG–vitronectin association is to allow the binding of a ternary complex composed of vitronectin, thrombin, and antithrombin to cell surface proteoglycans to facilitate internalization and degradation of the complex.^77^ In disease, the increased concentration of vitronectin would increase this degradation and thus decrease thrombin and antithrombin availability.

As with vitronectin, fibronectin is abundant in plasma and is a major component of the ESL.^78^ It is involved in numerous cellular processes, including development, organogenesis, cell adhesion and migration, hemostasis, angiogenesis, and vascular remodeling.^78^ Plasma fibronectin circulates in the blood in a compact conformation until it binds to endothelial GAGs.^78^ It then alters its structure to form an extended conformation that subsequently assembles into fibrils.^78^ A consequence of this binding is that plasma fibronectin interferes with antithrombin binding to immobilized LMWH; however, antithrombin is only completely displaced from heparin at fibronectin/antithrombin ratios greater than those found physiologically.^39,79^ On the other hand, the binding of injected heparin to endothelium-bound fibronectin fibers induces a conformational change in fibronectin that increases its affinity for vascular endothelial growth factor.^80^

Fibrinogen is another abundant plasma protein and is the main protein responsible for the creation of blood clots. Fibrinogen binds to endothelial cells through surface proteoglycans, which facilitates clot formation.^81^ Heparin binding to this bound fibrinogen (to which they have a higher affinity than to free fibrinogen) then mitigates clot nucleation through the formation of a fibrinogen–heparin–thrombin ternary complex.^81^ As a consequence of this binding, fibrinogen is also involved in anticoagulant GAG neutralization. The direct study of fibrinogen-mediated GAG neutralization is complicated by the fact that thrombin cleaves fibrinogen into fibrin. Yet, fibrinogen is known to be more effective at neutralizing DS than both PF4 and histidine-rich glycoprotein (HRG).^58^ This neutralization occurs at physiological fibrinogen concentrations, and the mechanism seems not to be through direct competition for DS binding but by modulating the rate of formation of the thrombin–HCII complex.^58^ Fibrin can form complexes with heparin, antithrombin, and thrombin to reduce thrombin inhibition by antithrombin.^59,60^ Because of those interactions, plasma fibrinogen levels are linked to heparin resistance in patients.^82^

In addition to those proteins, both Ca^2+^ and Zn^2+^ are released from activated platelets, and they can also affect the activity of GAGs.^83^ The role of Zn^2+^ in the neutralization is particularly interesting as the concentration of labile Zn^2+^ in plasma can be directly influenced by free fatty acid levels in plasma through a switch on human serum albumin, the main plasma transporter for both Zn^2+^ and free fatty acids.^84,85^ This dynamic may be important for individuals with diabetes mellitus,^86^ obesity,^87^ and cancer^88^ who typically associate with higher plasma free fatty acids levels and have a higher incidence of developing thrombotic complications.^89^ This is further supported by a study suggesting that higher doses of UFH are required in diabetic versus nondiabetic individuals.^90^

## GAG Neutralization During Inflammation

Inflammation and coagulation are processes that are closely linked because such inflammatory proteins often come into contact with GAGs and can influence their anticoagulant activity. Some proteins even play a dual role in both processes, as is the case of HRG, a key adaptor protein released by platelets that regulates angiogenesis, immune functioning, and coagulation.^91,92^ HRG is the second most abundant HS-binding protein in plasma after antithrombin and binds endothelial HS in a Zn^2+^-dependent manner. HRG–GAG binding is thus enhanced at injury sites where platelets release Zn^2+^.^62^ This allows the protein to both neutralize anticoagulant GAGs and to provide a tether site on the ESL to facilitate interaction with ligands such as plasminogen.^85,93^ In addition, HRG can compete with FGF for binding to HS and thus mediate the mitogenic activity of growth factors.^94^ High-molecular-weight kininogen (HMWK) and serum amyloid P protein share similar heparin-neutralizing functions (and ligands-binding properties) to HRG and are present in plasma in similarly high concentrations.^64,65^ HMWK is involved in coagulation through the activation of factor XII. During this action, HMWK is cleaved by kallikrein into the peptide bradykinin, which plays a role in vasodilation.^64^ Serum amyloid P protein is involved in the innate immune system and in clearing cellular debris but also contributes to the progression of neurodegeneration through its interaction with amyloid fibers.^44,95^ HRG, HMWK, and serum amyloid P protein can all bind to polyanions such as GAGs but also pathogens, anionic phospholipids (such as those exposed by dying cells), and DNA.^94^ For certain pathogens, this interaction can destabilize the membrane, leading to cell death or can reduce their pathogenicity through incorporation of the organisms inside fibrin clots.^94^ Similarly, HRG can also tether IgG to necrotic cells through binding to specific phospholipids at their surface and thus facilitate their phagocytosis.^96^ Binding of HMWK to polyanionic surface exposed by damaged cells also activates the kallikrein coagulation pathway.^94^

Lactoferrin is a low abundance (<19 nmol/L) iron-binding plasma protein stored in neutrophils.^97,98^ It can increase in concentration up to 3-fold during severe infection, autoimmune disease, or pregnancy, in addition to a local increase at the site of infection.^47^ It has been shown to bind and neutralize heparin in a dose-dependent manner, and its activity is comparable to that of PF4.^66,99^ However, this interaction can outcompete the binding of pathogens at the cell surface, preventing cell entry and stopping infection at an early stage.^100^ Thus, lactoferrin displaced by exogenous heparin administration has the potential to negate this activity and leave the organism more vulnerable to infection. Another inflammation-associated protein is alpha-1-acid glycoprotein, an acute-phase protein responsible for modulating the immune response. It inhibits platelet aggregation and is an important plasma drug carrier involved in transporting heparin.^48^ Alpha-1-acid glycoprotein is abundant in plasma and is upregulated in certain disease states (liver cancer, HIV infection), drug use, or pregnancy. In addition to its basal concentration in plasma, it is also secreted locally by activated neutrophils.^48^ Within the ESL, it plays an important role in maintaining capillary permeability.^101^ Alpha-1-acid glycoprotein can neutralize heparin but only when present at high concentrations, such as those that occur during inflammation.^67,68^ An injection of heparin could potentially saturate the transport site on the protein and prevent it from carrying other drugs or molecules.

Histones are usually associated with DNA inside cell nuclei but are released into plasma by activated inflammatory cells (to form neutrophil extracellular traps) and after cell death.^49^ They are mediators of cytotoxicity and sepsis during which their plasma concentration increases significantly.^102^ Histones have various procoagulatory activities (activation of platelets, stimulation of thrombin generation, and promotion of von Willebrand factor release), which include the ability to neutralize heparin.^69^ As a consequence, heparin–histone binding interferes with formation of neutrophil extracellular traps and perturbs venous thrombosis.^103^ Histones, however, bind more readily to other polyanions. Its interaction with polysialic acid, for example, is important in the development and regeneration of the nervous system.^104,105^

Although not strictly a GAG-neutralizing protein, kallistatin is a GAG-binding serpin. The binding of GAG prevents kallistatin from binding and inhibiting kallikrein, thus allowing activation of factor XII and cleavage of HMWK into bradykinin. Both events result in antiangiogenic and procoagulatory effects.^45,106^ An important consequence of this is that competition between kallistatin and vascular endothelial growth factor and bFGF for endothelial GAG binding reduces the angiogenic effects associated with these molecules.^106^

## Other Molecules That Can Neutralize GAGs

Lipoproteins are macromolecular complexes made up of lipids and protein. Depending on their size, they are classified as chylomicrons, very-low-density lipoproteins, intermediate-density lipoproteins, low-density lipoproteins, and high-density lipoproteins.^107^ In the blood, they are involved in the transport of cholesterol, triglycerides, and fat-soluble vitamins.^107^ They are therefore present in the blood and interact easily with GAGs. Low-density lipoproteins form insoluble complexes with heparins.^108,109^ Very-low-density lipoproteins have a similar but reduced effect on heparin while high-density lipoproteins do not neutralize heparin.^70^ The binding of lipoproteins to endothelial proteoglycans and the subsequent inflammatory responses could potentially have important consequences in the initiation and progression of the atherosclerotic process.^13^ Other proteins have also been shown to neutralize heparin (tissue factor,^71^ factor VII,^72,73^ and the axon guidance protein, Slit3^74^); however, their low concentrations make them unlikely to play a major role in endogenous neutralization.

## Breakdown or Downregulation of GAGs

In addition to their neutralization, heparin, HS, and DS can be prevented from exercising their anticoagulant actions through either a reduction in synthesis or through targeted degradation. Homocysteine, a compound generated during amino acid synthesis, is an important regulator of GAG synthesis.^110^ Its main action is to inhibit the protein C anticoagulant pathway by decreasing the thrombomodulin pool at the surface of endothelial cells and reducing protein C activation.^111^ In addition, homocysteine has been shown to diminish the synthesis of anticoagulant HS at the surface of endothelial cells, thus also reducing the antithrombin-binding HS pool.^111^ This process occurs at a slower rate than the inhibition of protein C and does not directly influence HS already present at the surface of the cells. To enable inhibition of HS synthesis, the homocysteine concentration needs to be around 100 μmol/L. This concentration can be achieved in vivo but only in certain disease states (eg, genetic polymorphisms in *MTHFR*—the gene encoding methylenetetrahydrofolate reductase, which is required for homocysteine synthesis) and in severe nutritional deficiency.^110,111^ However, a concentration of 10 μmol/L may be sufficient if the redox potential of the cell is influenced by other factors, for example, by the presence of certain cations such as Cu^+^, Cu^2+^, Fe^2+^, or Fe^3+^.^110,111^ Such elevated homocysteine levels are associated with cardiovascular diseases, disorders associated with abnormal renal function, administration of certain lipid-lowering drugs, and also caffeine or alcohol consumption.^110,111^

More directly, anticoagulant GAGs can be degraded by lyases (heparinases) or hydrolases (heparanases and elastases). Such enzymes are released during inflammation from macrophages after they are activated. These include cathepsin S, which can directly hydrolyse GAGs.^112^ Antithrombin and HCII can also be cleaved by neutrophil elastase or by cathepsin G. These enzymes are present in the primary granules of neutrophils and are released during inflammation. Their cleavage of antithrombin and HCII is enhanced by the presence of GAGs.^113–115^

## Summary and Clinical Impacts

There are multiple proteins that impact on coagulation via GAG neutralization to a degree which is not fully appreciated. These include proteins involved in control of inflammation, lipid transport, and cellular communication. In addition, most of the molecules that bind GAGs (many of which were not highlighted in this review) in vitro are unlikely to do so under physiological conditions where protein interactions are more complex (ie, presence of multiple interacting partners, formation of ternary complexes). Clarification of which GAG-binding proteins are relevant in vivo is thus still required. Details of the neutralization mechanisms involving HS and DS are lacking. Binding and neutralization of cell-associated HS and DS by proteins are more complex to study than with heparin, and this difficulty has likely limited the information available on their interactions with proteins. Furthermore, many studies examining GAG–protein interactions have focused on the resultant impact on other physiological processes and not coagulation, and so further studies are required to uncover specific roles in neutralization of anticoagulant GAGs. Finally, the relevance of endogenous heparin to physiological coagulation control is still controversial as genetic studies supporting its lack of importance (*NDST2* knockout mice) did not specifically analyze clot parameters.^21,22^

It is clear that the wide range of proteins that influence the anticoagulant properties of GAGs will affect a patients’ response to particular forms of heparin or heparin-based drugs. Indeed, levels of GAG-neutralizing proteins are influenced by an individual’s genetics, age, diet, and disease state. In a clinical context, this makes the dose–response profiles of heparins and heparin-based drugs difficult to predict. Knowing more about endogenous molecules that bind to GAGs and those that regulate their turnover will enable a better understanding of clotting disorders and treatment choices. Personalized treatments taking into consideration the plasma levels of a particular neutralizing protein may also be considered. In addition, it is important to take those information into account when dealing with specific disorders such as heparin-induced thrombocytopenia or pathologically high zinc plasma levels. More trials are needed in this area to better understand the advantages and drawbacks of each GAG when given to particular subsets of patients. Current guidelines also vary widely between regions. Consistency and better application of these guidelines is required by hospitals to provide a better care to patients. New knowledge gained by studying GAG neutralization will also aid the development and application of new clinical heparin neutralizers. Indeed, as protamine sulfate treatment is known to cause several adverse effects, including anaphylaxis, hypertension, nausea/fatigue, and back pain,^27^ other proteins or molecules including heparin-binding synthetic peptides are already being trialed as potential replacements.^4^

## Sources of Funding

This work was supported by the British Heart Foundation (grant codes: PG/15/9/31270 and FS/15/42/31556). S.J. Pitt is supported by a Royal Society of Edinburgh Biomedical Fellowship (XRE013).

## Disclosures

None.

## Notes

Highlights

- The glycosaminoglycans, heparan sulfate, dermatan sulfate, and heparin are important anticoagulants that inhibit clot formation through interactions with antithrombin and heparin cofactor II.
- Unfractionated heparin, low-molecular-weight heparin, and heparin-derived drugs are used clinically to treat coagulatory disorders.
- A wide range of proteins have been reported to bind and neutralize glycosaminoglycans, as reviewed here.
- The anticoagulant activity of glycosaminoglycans may also be regulated through inhibition of synthesis or by degradative enzymes during inflammatory processes.
